# Comparison of self-esteem and depression among fertile and infertile women

**DOI:** 10.1192/bjo.2021.176

**Published:** 2021-06-18

**Authors:** Akani Ruth, Akinyemi Akintayo

**Affiliations:** 1 Lincolnshire partnership NHS foundation trust; 2 Piedmont Athens regional medical center

## Abstract

**Aims:**

This study aimed to explore the effect of infertility on self–esteem and depression, and to identify the sociodemographic and infertility characteristics associated with self-esteem and depression among infertile women in Ekiti State, Nigeria.

**Method:**

Self-esteem and depression were assessed in 100 infertile women and 100 women seeking family planning (controls) using the Rosenberg Self-Esteem Scale (RSES) and Patient Health Questionnaire (PHQ-9), respectively. PHQ-9 score ≥10 was defined as major depressive disorder (MDD). Continuous variables were presented as mean ± standard deviation and categorical variables as frequency (percentage). Cases and controls were compared using Student's t test. χ2 or Fisher's exact (when cell size <5) tests were performed to compare proportions. Simple and multiple linear regression analyses were used to examine the association between the sociodemographic, infertility characteristics and RSES or PHQ-9 scores among infertile women

**Result:**

Infertile women had significantly lower RSES score (19.4 ± 4.5 vs. 20.7 ± 4.4, p = 0.038) and higher PHQ-9 score (5.1 ± 4.1 vs 3.8 ± 3.5, p = 0.023) compared to controls. Seventeen infertile women (17%) and 8 women in the control group (8%) had MDD (PHQ-9 score ≥10) and were referred for further evaluation. Among infertile women, marital status, being remarried, duration of infertility, and RSES score were associated with PHQ-9 score on simple linear regression. Similar association was not seen in the controls. On multiple linear regression analysis, RSES score had a negative association with PHQ-9 score (β = -0.32, p < 0.001) among infertile women. Older age [OR (95% CI):1.13 (1.01–1.25); p = 0.030], ≤6 years formal education [OR (95% CI): 4.76 (1.13–20.00); p = 0.033], being remarried [OR (95% CI): 10.87 (1.86–63.64); p = 0.008], longer duration of infertility [OR (95% CI): 1.11 (1.01–1.22); p = 0.040] and RSES score [OR (95% CI): 0.79 (0.67–0.92); p = 0.003] were significantly associated with MDD. On multiple logistic regression analysis, only the association between RSES score and MDD remained statistically significant (p = 0.004)

**Conclusion:**

Infertile women have lower self-esteem and higher depression scores in comparison to women seeking family planning. Mental health screening and management should be an integral part of care administered to infertile women

